# Active Inference in Psychology and Psychiatry: Progress to Date?

**DOI:** 10.3390/e26100833

**Published:** 2024-09-30

**Authors:** Paul B. Badcock, Christopher G. Davey

**Affiliations:** 1 Centre for Youth Mental Health, The University of Melbourne, Melbourne, VIC 3052, Australia; 2 Orygen, Melbourne, VIC 3052, Australia; 3 Department of Psychiatry, The University of Melbourne, Melbourne, VIC 3010, Australia; c.davey@unimelb.edu.au

**Keywords:** active inference, evolutionary psychology, depression, free energy principle, psychiatry, psychology

## Abstract

The free energy principle is a formal theory of adaptive self-organising systems that emerged from statistical thermodynamics, machine learning and theoretical neuroscience and has since been translated into biologically plausible ‘process theories’ of cognition and behaviour, which fall under the banner of ‘active inference’. Despite the promise this theory holds for theorising, research and practical applications in psychology and psychiatry, its impact on these disciplines has only now begun to bear fruit. The aim of this treatment is to consider the extent to which active inference has informed theoretical progress in psychology, before exploring its contributions to our understanding and treatment of psychopathology. Despite facing persistent translational obstacles, progress suggests that active inference has the potential to become a new paradigm that promises to unite psychology’s subdisciplines, while readily incorporating the traditionally competing paradigms of evolutionary and developmental psychology. To date, however, progress towards this end has been slow. Meanwhile, the main outstanding question is whether this theory will make a positive difference through applications in clinical psychology, and its sister discipline of psychiatry.

## 1. Introduction

Arguably, the aim of psychology is ultimately twofold. The first of these aims is to understand people. The second is to help them. In principle, these two goals are highly complementary, although in practice, the degree to which progress towards the former translates into progress toward the latter is open to question. Although we will revisit this issue later, the aim of this review is to explore the extent to which the active inference framework has contributed meaningfully to both of these aims, and in so doing, informs the twin disciplines of psychology and psychiatry. A review of the growing body of work that has sprung up around active inference in the past decade or so is beyond the scope of this article, although it must be said that its influence on psychology remains curiously nascent. With this in mind, we will explore its promise and challenges by considering its application to two areas of our own interest—namely, global theories of the form and function of the brain (i.e., an attempt to understand people); and theories of depression, which stand to inform best-practice approaches to prevention and intervention (i.e., an attempt to help people). By considering its contributions to progress in both of these fields, we hope to show that the theory of active inference has the clear potential to unify theorising and research in psychology as a discipline, before turning to advances in clinical psychology and psychiatry, which are best positioned to translate this theory into practical applications that help those in need.

## 2. Understanding the Psyche

**Figure 1:**
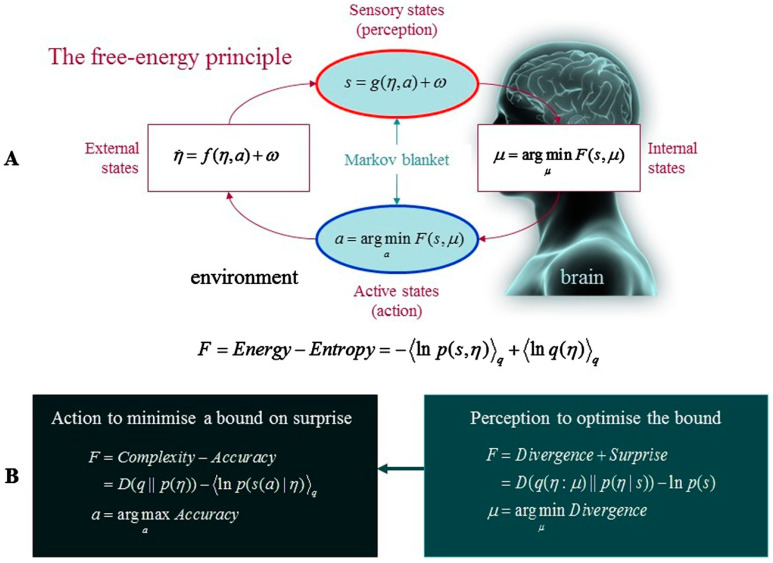
**The free energy principle**. (**A**): Schematic of the quantities that define free energy, including the internal states of a system, *μ* (e.g., a brain), and quantities that entail the system’s exchanges with the environment; including its sensory input, *s* = *g*(*η*,*a*) + *ω*, and actions, *a*, which change the ways in which the system samples its environment. Here, environmental states are described by equations of motion, *η*˙ = *f*(*η*,*a*) + *ω*, which capture the dynamics of (hidden) states extraneous to the system, *η*, while *ω* refers to random fluctuations. Under this scheme, internal states and action work in tandem to minimise free energy, which reflects a function of sensory input and the probabilistic representation (variational density), *q*(*η*:*μ*), that internal states encode. External and internal states are also statistically separated by a Markov blanket, which entails both ‘sensory’ states and ‘active’ states. Internal states are affected by, but cannot influence, sensory states, while external states are affected by, but cannot influence, active states, which creates a conditional independence between the system and its environment. (**B**): Alternative equations that capture the minimisation of free energy. With respect to action, free energy can only be reduced by the system’s selective sampling of (predicted) sensory input, increasing the accuracy of its predictions. Conversely, optimising internal states (i.e., perception) minimises divergence by rendering the representation an approximate conditional density on the hidden causes of sensory input. This reduces the free energy bound on surprise, which means that the system can avoid surprising sensations through action. Reprinted from “Answering Schrödinger’s question: A free-energy formulation”, by Ramstead et al. [60] (p. 4). Copyright 2018 by Elsevier.

Given the wealth of papers that describe how active inference manifests in the brain and our behaviour, e.g., [1,2,3], we will not revisit this work here. Put briefly, this model suggests that living systems are able to actively avoid decay by generating predictive, self-fulfilling action–perception cycles that ensure we remain within a limited range of unsurprising phenotypic states (see Figure 1). Having said that, we will now concentrate on how this framework provides a compelling solution to long-standing debates in psychology about the ways in which the brain is structured and functions, and the corresponding mechanics of human development and biobehaviour. To appreciate the potential impact of Friston’s work on our understanding of how the mind works, it is worth revisiting the state of play in psychology when his free energy principle first entered the theoretical landscape. 

For many years, proponents of global theories of brain function in psychology were divided into two camps. On the one hand, there were *evolutionary psychologists*, many of whom remain staunch advocates of *massive modularity*, which suggests that the brain comprises a large collection of functionally specialised modules that have been designed by natural selection to solve specific adaptive problems [4,5,6]. This model places strong emphasis on the *domain specificity* of neural subsystems, and the influence of natural selection and the ancestral environment on how the contemporary brain is structured and functions. As a research program, this model translates into two main levels of inquiry. Following Marr [7], one first needs to attend to the *computational* level of analysis by identifying the adaptive problem in the ancestral environment that a given trait has been designed by selection to solve; one must then address the *algorithmic* level of explanation by identifying the various (environmental and physiological) inputs and (cognitive and behavioural) outputs that enable the trait to solve that adaptive problem [8,9]. 

This approach has been criticised for promoting human essentialism and genetic determinism, as well as its failure to account for neural plasticity, individual differences, and domain-general processes [10,11,12,13]. Not unfairly, the massive modularity hypothesis has itself been criticised for pitching a naïve and neurobiologically implausible model of the brain [10,14,15,16,17]. For this reason, some evolutionary psychologists have turned towards more sophisticated network-based models of brain function, e.g., [18,19,20]. It should also be recognised that the utility of evolutionary psychology as a research heuristic largely rests on the *phenotypic gambit*, which allows us to analyse the adaptive function of a given trait without requiring an understanding of the neurobiological mechanisms responsible for producing it [21,22]. This points to the importance of distinguishing between massive modularity as an explanatory claim about the form and function of the brain and its clear value as a research heuristic [16]. Regardless of *how* the brain is structured and functions, natural selection and other evolutionary forces are key to understanding *why* we think and act in the ways that we do [23,24,25,26,27,28]. 

Meanwhile, the other side of the meta-theoretical divide has been dominated by developmental psychologists, who maintain that the structure of the brain emerges from the experience-dependent self-organisation of neural circuits over time [29,30]. Unlike massive modularity, this view suggests that infants only begin with a highly limited set of innate, genetically-specified predispositions, which allows recursive interactions between these lower-level systems and the environment to produce the functional organisation of the brain and its highly flexible, domain-general capacities over the course of ontogeny [31]. Central to this view is the theory of *self-organisation*, which refers to the emergence of stable, higher-order functional patterns from dynamical interactions between multiple lower-order components intrinsic to a dynamical system [32,33,34]. Although this developmental approach can be commended for its neurobiological plausibility, its clear neglect of Darwinian dynamics runs the risk of robbing theorists in the field of a fully accepted explanation for the adaptive properties of all biological systems, humans or otherwise [6,23,35,36]. 

Developmental approaches are well positioned to tell us how phenotypic traits emerge over the life course, but they are less equipped to answer ultimate questions about why such traits emerge, particularly those that are evidently species-typical, cross-cultural, reliably transmitted across successive generations, and demonstrably adaptive. That notwithstanding, there has been ample debate about the relative merits and pitfalls of both schools of thought for many years now (e.g., [4,37,38,39]), so we will not delve into these here. Suffice it to say, there remains staunch disagreement between those who argue that natural selection is the principal mechanism driving human cognition and behaviour, and those who champion the causal primacy of self-organisation and developmental constructionism, robbing psychology of a clear consensus regarding a unified global brain theory to unite its subdisciplines.

This is not to say that attempts have not been made to synthesise the two. In particular, proponents of evolutionary developmental biology (and its sister discipline of evolutionary developmental psychology [40,41]) have forwarded *epigenetic modes of inheritance* as an adaptive, intermediary step between evolution and development, such as cellular epigenetic inheritance, socially mediated learning, and for humans, symbol-based information transmission [42,43,44]. These allow adaptive behaviours and phenotypic modifications to be transmitted to offspring without directly altering the genome, thereby supplying new targets for selection. Similarly, others have emphasised the key role that culture plays in shaping human phenotypes over evolutionary time [45,46,47,48,49]. By concentrating their critiques upon the ‘Sante Barbara school’ of evolutionary psychology, and its commitment to the massive modularity hypothesis, developmentalists and other critics have tended to overlook the broader paradigm of evolutionary psychology, which embraces the synergistic relationship between evolutionary and developmental processes, while still attributing causal primary to the influence of selection [23]. 

In this vein, others have also argued that natural selection and self-organisation are best seen as commensurate and complementary (e.g., [33,50,51,52]). This *evolutionary systems* perspective suggests that in order to explain a given trait, one needs to combine an ultimate analysis with a proximate one—a Darwinian approach is required to explain *why* a particular phenotypic trait has evolved, while self-organisation allows us to explain *how* it emerges and operates [23,51]. To date, evolutionary systems theorists have largely focused on *complex adaptive systems*, which—like the brain—adapt to the environment through a process of selection that recruits the outcomes of locally interacting components within that system to select a subset for replication or enhancement [53]. Under this view, Darwinian principles can be reframed as a dynamical process of variation-selection-retention that pervades all self-organising systems [50,54,55,56].

Altogether, there is good reason to believe that metatheoretical tensions in psychology can be resolved by adopting an integrative, evolutionary systems approach to understanding human phenotypes, which weds theorising in evolutionary and developmental psychology to explain the brain and our behaviour across temporal and spatial scales. One way to operationalise this approach is to organise different paradigms in psychology according to Tinbergen’s seminal four levels of biological inquiry: *adaptive function*, *phylogeny*, *ontogeny*, and *mechanism* [57]. Each of these levels involves both a *temporal* dimension (ranging from evolutionary time through to real-time processes) and a *systemic* dimension (ranging from all *Homo sapiens* to an individual *in situ*). Here, different paradigms are seen to be complementary, because they concentrate differentially on four interrelated levels of analysis—functional hypotheses for adaptive, species-typical traits (e.g., evolutionary psychology); explanations for intergenerational, phylogenetic processes that drive evolutionary change and underpin our flexible adaptation to local eco-niches (e.g., evolutionary developmental biology and psychology); explanations for individual development (e.g., developmental psychology); and mechanistic explanations for real-time biobehavioral phenomena, which roughly fall under the purview of the sub-disciplines [23,58]. Although they are often pitched against each other, these different levels of analysis should instead be treated as distinct, alternative and valid perspectives of the same whole [59]. Meanwhile, the non-substantive meta-theory of evolutionary systems theory, which centres on the dynamic interplay between selection and self-organisation, is capable of synthesising theorising across the psychological sciences by encapsulating all four of Tinbergen’s questions [18,23,60]. 

Although this perspective makes sense of the meta-theoretical structure of psychological science, its plausibility also rests on finding a compatible, evidence-based theory of the brain. However, at the time active inference first appeared in the psychological literature, both major camps in psychology continued to disagree on a suitable candidate. Nevertheless, there were still some emerging points of agreement about the brain’s architectural properties. Both developmental and evolutionary psychologists converged on the idea that the lowest or most peripheral levels of the cortical hierarchy comprised relatively *segregated*, highly specialised neurocognitive mechanisms responsible for sensorimotor processing (so called ‘domain-specific’ systems), while its higher, deeper or more central layers consisted of developmentally plastic, highly *integrated* (‘domain-general’) mechanisms [27,61]. These are widely distributed subsystems that respond flexibly to input received from multiple lower levels, feed information downstream for further processing, and underlie the executive cognitive functions unique to humans [18,62]. 

There are intersecting strands of theory and evidence to support this view. For example, large meta-analyses of task-based neuroimaging data provide evidence for domain general systems, by showing that distinct regions of the brain have different functional partners in different contexts (e.g., [10,11]). Similarly, responses to unimodal sensory input have been found to be affected by information processed by other sensory modalities, with latencies suggesting that inputs in one modality directly influence early responses to stimuli presented to another [63]. This provides clear evidence of functional integration, even at the level of the sensorium. Conversely, high-resolution structural connectivity findings have found that specialised motor tasks have a distinct, structural (segregated or modular) counterpart, providing evidence of domain specificity [64,65,66]. 

Focusing now on the brain’s hierarchical structure, wide-ranging studies of structural and functional connectivity have confirmed that the brain instantiates a *self-similar hierarchy*, where a given node comprises a network of smaller interacting nodes at a lower hierarchical level, ranging from macroscopic brain regions down to neurons [65,67,68,69]. Consistent with this, both comparative and human studies have shown that a hierarchical structure is a hallmark of the mammalian brain, which mirrors phylogeny by progressing from highly segregated sensorimotor hierarchies found in all mammals through to the more recent, highly distributed association areas found in primates [14,70]. On the one hand, natural selection ensures the development of highly specialised sensorimotor networks in infancy, which function as ‘neurodevelopmental anchors’ that allow the progressive development of domain-general association regions [62,71]. On the other hand, these association regions enhance evolvability by allowing us to respond flexibly to rapidly changing environments. Others have presented evidence that the structure of the brain echoes the complementary relationship between evolution and development (e.g., [14]), with different levels of Tinbergian timescales reflected across nested levels of the brain—ranging from the genes inherited from our ancestors; to the epigenetic transcription factors that shape gene expression; to the epigenesis of neural networks over development; and ending with the long-range connections that underwrite daily consciousness [72]. 

Crucially, the brain’s hierarchical structure also replicates the hierarchically nested structure of causal regularities in the environment, with its lower, more peripheral layers encoding rapid environmental fluctuations associated with sensorimotor processing and stochastic effects, while its higher, more central layers encode increasingly slower regularities related to longer-term contextual changes [73,74,75,76]. A hierarchical organisation is also a key property of complex adaptive systems [77,78]—it enhances evolvability by extending the parameter range for self-organised criticality, a dynamical state that optimises information processing and is therefore favoured by selection [79]. Notably, it also enables a system to solve problems by recursively combining solutions to subproblems, with simulation studies of evolving networks showing that a hierarchical structure adapts faster to new environments than non-hierarchical ones [80]. 

Such findings notwithstanding, psychological scientists from both sides of the meta-theoretical divide were struggling to agree upon a global theory of the brain that was able to reconcile their debates about the causal primacy of evolutionary versus developmental processes. This underscored the need to seek a suitable candidate in neuroscience. 

## 3. Active Inference: A New Direction for Psychological Science?

Although the idea had been brewing in the literature for some time (e.g., [2,81,82]), the publication of Friston’s [1] outline of the free energy principle and the active inference framework proffered a genuine solution to the meta-theoretical stalemate that has long plagued psychology. To begin, its alignment with *predictive processing* in neuroscience supplied an empirically robust *process theory* that could explain the hierarchical structure and functioning of the brain. Briefly, this suggests that the brain reflects a hierarchical *inference machine* that reduces uncertainty about the causes of sensory input by minimising its prediction errors (see [83,84]). To do this, the brain attempts to optimise its predictions about the world by minimising discrepancies between incoming sensory inputs and top-down, neuronally encoded predictions. Prediction errors are also weighted by their *precision*, which relates to the reliability afforded to various beliefs or sources of sensory evidence, and involves neuromodulatory mechanisms (e.g., affecting attentional selection) that determine the relative influence of ascending (error) vs. descending (representation) signals on belief-updating [85,86]. Although this predictive processing account provides an empirically verifiable and strongly supported theory of the structure and functioning of the brain, it must be said that its interest is limited for many psychologists, who are more concerned with our behaviour.

Here is where the explanatory scope of active inference comes into its own. It not only supplies cognitive and behavioural scientists with a formally expressible and empirically testable model of neurocognitive processing, it also explains how such processes relate to action (and indeed, vice versa). In a nutshell, the idea that everything we think and do arises from the need to optimise our predictions about the world and behave in ways that fulfil them is elegant and profound [18,87]. Of particular interest to evolutionary psychologists, it is also clear that the theory accommodates Darwinian processes. Echoing Schrödinger [88], active inference rests on the observation that all living systems preserve their integrity by actively revisiting a small number of (expected and highly probable) phenotypic states. Unexpected or ‘surprising’ states, which are incongruous with the characteristic states of the organism, must be avoided in order to resist thermodynamic decay—take the prototypical example of a fish out of water [1]. Thus, an organism’s evolutionary drive to maintain survival by sustaining functional physiological states (i.e., homeostasis and allostasis) translates into a proximal avoidance of surprising states [1]. In line with evolutionary systems theory, the biological imperative to avoid such states is an outcome of Darwinian processes: self-organising systems that could resist thermodynamic decay by actively avoiding surprising phase-transitions have been favoured by natural selection [2]. Natural selection reduces surprise by endowing a small number of attractive (i.e., adaptive) states with innate value, which minimises surprise by ensuring an organism seeks out expected states that are consistent with its phenotype and environment [1,89]. Natural selection can therefore be seen as a process of Bayesian model selection that minimises the free energy of different species’ phenotypes (i.e., *generative models*; [55,60]).

**Figure 2:**
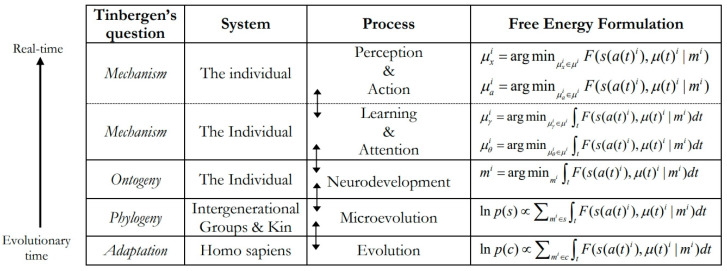
**The Hierarchically Mechanistic Mind**. The human brain instantiates an evolved and embodied complex adaptive system (i.e., a *generative model*) that actively minimises variational free-energy by generating self-fulfilling action–perception cycles, which are mediated by dynamic interactions between hierarchically organised neurocognitive subsystems [18]. This system emerges from causal interactions between ecobiopsychosocial dynamics that unfold across various spatiotemporal scales. Formally, these dynamics can be described in terms of the timescales over which free-energy minimisation optimises the state (i.e., *perception*), configuration (i.e., *action*), connectivity (i.e., *learning and attention*), anatomy (i.e., *neurodevelopment*), and phenotypes (i.e., *neural (micro)evolution*) of biological agents that belong to a given class or species (e.g., *Homo sapiens*). Under this formalism, $F(s(a^{i}),\mu^{i}|m^{i})$ reflects the free energy of the (action dependent) sensory data, s(a(t)), and states, *μ,* of the *i*-th agent, $m^{i}\in s$, that belongs to a subgroup, s∈c, of class, *c*. Action, *a*, governs the sampling of sensory data, while the physical states of the phenotype, μ, encode (probabilistic or Bayesian) beliefs and accompanying expectations (i.e., means or averages). In real time, neurocognition entails two dynamically coupled processes. The first, which encompasses *perception* and *action*, optimises neuronal and effector dynamics to attune the organism to its environment, by minimising prediction errors (resp. free energy) based on a generative model of the hidden causes of sensory data. The second process, which relates to *learning* and *attention*, optimises synaptic strength and efficiency over seconds to hours to encode the precision of prediction errors and the causal structure of the environment in the sensorium. Over the course of development, human generative models are optimised by activity-dependent pruning and the maintenance of brain structures and connections that are transmitted (epi)genetically. Over longer timescales, average free energy is optimised over generations of individuals who belong to a particular subgroup (e.g., kin) of a given class (i.e., conspecifics), through the intergenerational transmission of adaptive priors. Finally, natural selection optimises the average free energy over time and individuals who belong to a given class (i.e., conspecifics) through the effects of selective pressure on their generative models. Reprinted from “The mechanics of evolution: Phylogeny, ontogeny, and adaptive priors”, by Badcock et al. [93] (p. 54). Copyright 2024 by Elsevier.

By leveraging these insights to combine progress in cognitive neuroscience with evolutionary and developmental perspectives in psychology, the brain can be reframed in terms of an evolved, *hierarchically mechanistic mind* (HMM): a (situated and embodied) complex adaptive system that actively minimises the variational free energy (and therefore entropy) of (far from equilibrium) phenotypic states via self-fulfilling action–perception cycles, which are mediated by dynamic interactions between hierarchically organised (functionally differentiated and differentially integrated) neurocognitive mechanisms [18,58]. This hypothesis connects most closely with evidence in network neuroscience that the brain instantiates a self-similar hierarchy [65,68,90], along with more recent claims in the active inference literature that the brain consists of hierarchically nested Markov blankets [91,92]. The HMM is distinctive, however, in that it places evolutionary dynamics front and centre by emphasising the causal role of species-typical *adaptive priors*—namely, neurophysiologically instantiated Bayesian beliefs about our characteristic phenotypic and environmental states that have been shaped by selection to ensure that our action–perception cycles keep us within the adaptive bounds of unsurprising states [18,58,93]. Such evolutionary priors arise from the reliable transmission of adaptive (surprise-reducing) policies from one generation to the next, extending from innate, genetically inherited priors sculpted by natural selection, through to developmentally-open prior expectations that depend on the repeated assembly of reliably recurrent ontogenetic resources that arise from reciprocal interactions between (epi)genetic processes canalised by natural selection, developmental processes that unfold over the life course and situational activities in species-typical, real-time environments [93]. In short, adaptive priors allow our generative models of the world to be sculpted adaptively by natural selection, reliably passed on from one generation to the next, and successively optimised by neurodevelopment and learning [18]. Meanwhile, the non-substantive process theory of active inference can be used to integrate ecobiopsychosocial dynamics across temporal and spatial scales, ranging from the evolution of our species through to the thoughts and actions of an individual in real time (see Figure 2). 

On such grounds, Friston has clearly brought to light a promising solution to debates surrounding the fundamentals of the mind and our behaviour that have been waged in psychology for decades. Unlike the massive modularity hypothesis, here was a model that provided evolutionary psychologists with a neurobiologically plausible theory of cognition and behaviour, which clearly allowed for adaptationism. It was also capable of incorporating phylogenetic processes—those intergenerational, between-group dynamics responsible for producing evolutionary novelties and change [93], which should satisfy evolutionary developmental psychologists. As a theory of biological self-organisation [60,94,95], it should also sit well with developmentalists, providing a readily generalisable principle to explain neurodevelopmental processes—namely, the optimisation of human generative models (i.e., embodied brains) through activity-dependent pruning and the maintenance of structures and connections in the brain that are transmitted (epi-)genetically. Finally, under simplifying statistical assumptions, the theory is reducible to a simple hypothesis that can be readily extended across psychology’s sub-disciplines: cognition and behaviour work in concert to optimise our predictions about the world and ensure that we fulfil them. Simply put, we all strive to minimise our uncertainty. 

Presumably, this should have inspired the rapid uptake of Friston’s ideas across the discipline and provided both evolutionary and developmental theorists with some much-needed common ground. To this day, however, psychology appears to remain mired in Kuhn’s *pre-paradigmatic* stage of scientific progress [96], largely owing to persistent divisions between evolutionary psychologists and proponents of the standard social science model [97]. Arguably, then, the discipline is still in need of a new paradigm that can bring these schools together. Although we believe that active inference is well equipped to satisfy this need, the question remains as to whether it will attract the attention it deserves. 

## 4. Active Inference in Psychology: Progress to Date?

There has been a rapid uptake of active inference in psychology over recent years, which is impressive given how recently the model has emerged in the literature. In little over a decade, this theory has grown from being an esoteric global brain theory in computational neuroscience to a productive and fully generalisable research heuristic for understanding our minds and behaviour. To cite just a few examples, researchers have already applied this framework to explain subjective psychological phenomena, ranging from emotional states [98,99,100,101], illusions [102,103], and perceptual awareness [104], through to consciousness itself [105,106,107,108,109]. Conceptually, it is also consonant with prevailing schools of thought across the subdisciplines, such as clinical, cognitive, behavioural, social, and ecological psychology, by furnishing the requisite theoretical tools to incorporate foundational theories like representationalism [58], reinforcement learning [110], environmental affordances [111,112], self vs. other representations [113,114], and both dyadic and cooperative communication [115,116]. This is just a snapshot—more in-depth summaries of progress in the field can be found in dedicated volumes on the application of active inference across the cognitive sciences [3,84,117]. 

Altogether, the literature to date suggests that active inference has the clear potential to synthesise theorising and research across psychology’s subdisciplines, while also providing an explanatory framework that is evidence-based, biologically plausible, and able to resolve polarising debates between evolutionary and developmental psychologists by furnishing an agnostic, formal model of biobehavioural processes that equally accommodates Darwinian and developmental dynamics. However, progress toward a paradigm shift has been disappointingly slow. To date, applications of active inference have been wide-ranging but piecemeal, and seldom grounded in empirical studies conducted in real world settings. Instead, the vast majority of work has relied on conceptual and computational models, which are only tested *in silico* [118,119]. As of last year, the number of empirical studies in the active inference literature had barely reached double figures, with most addressing research questions in computational psychiatry instead of testing unique predictions derived from active inference itself [118].

Meanwhile, few if any of the major players in evolutionary and developmental psychology have adopted active inference. Luminaries responsible for establishing evolutionary psychology as a paradigm in the early 1990s have yet to take up this theory, and even the latest generation of influential thinkers in the field have neglected its clear synergies with adaptationism. Seminal thinkers in developmental psychology have also overlooked its explanatory scope, despite its emphasis on developmental self-organisation. Even leading developmentalists poised at the forefront of the intersection between psychology and neuroscience are sceptical of global brain theories such as predictive coding and active inference, e.g., [10,11], regardless of their impressive empirical support. More surprisingly still, advocates of evolutionary systems theory have remained curiously silent on the explanatory fruits of this theory, despite its emphasis on Darwinian dynamics operating upon self-organising systems [60,94,120,121]. 

This is not to say that such ignorance only goes one way. The influence of Darwinian dynamics and the key role played by adaptive priors in generating the form and function of living systems is decidedly under researched in the active inference literature. Beyond a smattering of our own contributions on adaptive priors [18,58,116,122,123,124], other work is limited, which tends to focus on formal models of evolutionary processes tested *in silico* (e.g., [55,120,121]). A clear shortfall of these models is that they overlook the importance of intergenerational dynamics (e.g., epigenetic inheritance) [125], which lie at the intersection between phylogeny and ontogeny, providing the grist for microevolution [93]. Nor are they tailored to model the sheer complexity of human systems. We are also unaware of any works in the field that have tried to capture the ways in which the environmentally nested biobehavioural patterns that unfold across the course of ontogeny reflect adaptive, recursive patterns of active inference over time. Admittedly, some theorists have applied the theory to development in utero [126] and attachment dynamics in infancy [127], but these models are rudimentary and go no further than the first steps on our ontogenetic journey. Curiously, researchers specialising in active inference have also yet to tackle personality differences—a fundamental source of study for evolutionary and developmental psychologists alike [128]. It is clear, then, that proponents of active inference and psychological scientists still have much to learn from each other. 

At this point, it should also be recognised that many of the key themes highlighted by active inference have been echoing through the chambers of psychology for over 50 years. Perhaps the most exemplary case is *control theory*, a model of self-regulating systems introduced to the field by Carver and Scheier in the 1980s. Inspired by Wiener’s seminal work on Cybernetics in 1948 [129], this paradigm hinges on the mechanism of *negative feedback loops*, which function to actively reduce perceived deviations from a given comparison value [130]. Under this framework, a system’s input function is the perception of a present condition, which is compared against an internal point of reference, or a *comparator* (e.g., goals). When discrepancies are perceived between the present state and the reference value, a behaviour is enacted (i.e., the output function), which aims to reduce the discrepancy between perceived and desired outcomes by changing the system’s environment [130]. Much like active inference, control theory suggest that cycles of action and perception create a closed *loop of control*—iterative comparisons between goals (e.g., adaptive priors) and environmental feedback determine cognitive activities that regulate actions, which serve to minimise deviations between current states and desired outcomes [131]. Notably, Carver and Sheier were also heavily influenced by Powers’ foundational work on what has widely become known as *perceptual control theory* [132], which has itself been forwarded as a unifying concept for psychology’s subdisciplines [133]. According to this view, behaviour is ultimately determined by the need to control perceptions—we constantly compare our actual perceptions against our desired perceptions or goals, and then act upon the world to achieve greater congruence between them. It goes without saying that both these schools of thought suit active inference to a T—an important parallel that has been discussed before (e.g., [106,134,135]). Akin to the HMM, they also both rest on the idea that control systems are inherently hierarchical: higher-level goals determine the comparators (sub-goals) for lower-level control systems, allowing for complex behaviour by coordinating the functioning of lower levels, with a view to achieve desired (adaptive) states [130,132,136].

Around the time cybernetics was introduced to the scientific community, there were other theoretical developments in psychology’s subdisciplines, which foreshadowed key principles that have since been espoused by the active inference community. Take, for example, ecological psychology, which stemmed from the foundational works of Gibson [137,138], Barker [139], and Bronfenbrenner [140,141]. This relational approach looks at the ways in which action and perception emerge from reciprocal organism–environment relationships over time [142]. Emphasis is placed on *environmental affordances*, which refer to mutually reinforcing dynamics between the abilities and expectations of an organism and aspects of its environment [138,143]. In line with active inference, ecological psychologists advocate a pragmatic, action-oriented approach to cognition, exploring the ways in which environmental affordances influence behavioural policy selection to achieve desired ends [142]. Around the same time, Bowlby—arguably one of the first evolutionary psychologists—proposed *attachment theory*, which suggests that early interactions between primary caregivers fundamentally shapes an individual’s expectations and behaviours in social relationships over the life course [144]. Subsequent work, first championed by Ainsworth, has evidenced the manifold ways in which secure, anxious, or avoidant attachment styles arise from attachment patterns observed in childhood, creating self-fulfilling cycles of relational expectations and behaviours that significantly impact later relationships and mental health [145,146]. Themes that are highly reminiscent of active inference had also cropped up in cognitive psychology, as exemplified by Rescorla’s work on prediction in learning theory [147,148]. Rescorla revised models of classical conditioning at the time by showing that the strength of a conditioned response relies upon the predictability of the conditioned stimulus, relative to the unconditioned stimulus. Contrary to his contemporaries, Rescorla described conditioning in terms of the learning of relationships between events to allow an organism to represent and predict its environment, underscoring the key role played by expectation and prediction in learning processes [147,149]. This mirrors active inference, of course, but also much broader ideas in cognitive science, extending from those initially developed by Helmholtz [150] through to contemporary advances in predictive processing [117]. 

There are no doubt other examples of subdisciplinary insights that pre-empted active inference—our brief review of the literature here is intended to be illustrative, not exhaustive. However, despite the emergence of these common themes at the time, such research remained highly fragmented, and psychological scientists were slow to join the dots. This is understandable—each of these camps suffered from the theoretical fragmentation that plagues psychology, obstructing cross-fertilisation and growth between different paradigms in the field [23,151]. Returning to our earlier point, the benefit of active inference is that it provides a clear, meta-theoretical solution to the disciplinary inertia that has long been driven by sub-disciplinary divisions. It does so by offering the requisite conceptual and methodological tools to generate and test hypotheses specific to different research domains, while providing a common language for meaningful collaboration and scientific advancement across the discipline of psychology itself. Indeed, it is worth noting that active inference has already been applied to the paradigms raised above, ranging from control theory [134,135] to the study of attachment dynamics [152] and the influence of environmental affordances on our predictions and behaviour [121]. This clearly speaks to its integrative power by evincing its capacity to incorporate and explain widely accepted phenomena highlighted by disparate paradigms. 

Altogether, we hope it is clear by now that active inference and major paradigms in psychology reflect two sides of the same coin. The former provides an evidence-based, non-substantive *process* theory of the form and functioning of the brain, while research in psychology helps us explain, substantively, both how and why humans think and act in the ways we do [18,58]. In practice, this calls for a dialectical relationship between computational and cognitive neuroscience, on the one hand, and research in psychology, on the other, favouring their mutual enlightenment by allowing insights gleaned from one to inform and constrain theorising and research in the other [21,153,154,155]. For neuroscientists, this requires experimental designs that can isolate the specific psychological factors that govern various patterns of hierarchical brain activity across different contexts. Examples include meta-analyses of task-based fMRI activation studies to characterise the functional fingerprints of particular brain regions across different tasks [10,11], and the development of *cognitive ontologies* that systematically map interrelations between particular cognitive functions and their corresponding patterns of brain dynamics [156,157,158]. Sophisticated longitudinal designs are also required, which combine neuroimaging studies on human brain maturation with biobehavioural and social measures to explore how different developmental contexts produce stable individual differences in perceptual biases and active inference [58,159,160]. Comparative, cross-cultural, computational, and dynamical approaches in evolutionary psychology are also necessary if active inference theorists wish to identify and unpack the particulars of our species-typical adaptive priors [58,161]. Finally, computational models and simulation studies enable us to model how different levels of dynamical activity interact [37,60,162,163], allowing researchers to explore how the biobehavioural phenomena described and studied by psychologists reflect adaptive free energy minimisation within and across spatiotemporal scales. The outcomes of such analyses can then be confirmed by experimental work and other real-world observations [18,161,164].

Conversely, and as we have stressed already, active inference offers a formal and empirically tractable *process theory* of the human brain, mind, and behaviour to psychologists. Although the mathematical apparatus that underwrites this theory is inaccessible to many [165,166], as we alluded to earlier, it can be translated more simply into an elegant heuristic that can be leveraged by researchers across psychology’s subdisciplines: cognition and behaviour work together to minimise our exposure to surprise [58]. Our lives reflect a dynamic, self-fulfilling prophecy of sorts—everything we think and do stems from the biological imperative to optimise our predictions about causal regularities in our eco-niche, and to behave in ways that confirm them [18,87,94,167]. Like others before us, we believe this elegant idea offers a promising common language to synthesise and explain diverse findings across the discipline of psychology. 

Obstructively, though, active inference is still plagued by translational issues, presumably arising from its origins in statistical thermodynamics and machine learning. This has led to concerted attempts to make its models and methods more accessible, such as step-by-step tutorials on how to apply active inference to real-world experimental settings (e.g., [3,166]). Such attempts have largely fallen on deaf ears, however, with little more than a handful of real-world empirical applications appearing in the literature to date [118]. Despite its clear explanatory scope, active inference remains largely neglected by psychological scientists, suggesting an element of institutional inertia that is typical of any discipline poised on the edge of a paradigm shift [96]. Still, as more come to understand this theory, and translate it into innovative empirical designs, one gets the sense that this paradigm shift is certainly plausible. Unfortunately, however, we suspect that this is still a long way off. As a whole, psychologists are highly sceptical of grand unifying theories [151], and outside of this field, active inference has received its fair share of critiques (e.g., [168,169,170,171,172]), which have yet to be fully addressed by its proponents. Such debates aside, our own view is that active inference deserves widespread advocacy, but the challenge remains to validate it empirically and meaningfully implement it in real-world settings. This returns us to the second main aim of psychology—the issue of whether active inference stands to help people. 

## 5. Active Inference in Clinical Psychology and Psychiatry: Is It Helping People?

Of all the subdisciplines of psychology, active inference has arguably borne the ripest fruit in clinical psychology, and even more so in its disciplinary sibling of psychiatry. This raises the second question driving our discussion—has the theory made significant inroads with respect to the second main goal of psychology: our attempts to help people? 

In the active inference literature, theories of psychopathology are typically framed in terms of false inference, e.g., in the case of hallucinations and delusions, inferring that something is there when it is not [173,174,175]. Although much of this work has focused on psychosis (e.g., [176,177,178,179]), here, we will concentrate on advances relating to depressive disorders—first, because this relates to our own field of research; and second, because the prevalence and disease burden of these disorders is so high that if we want to help people, concentrating on depression is clearly a priority. How, then, can depression be framed in terms of false inference? 

Broadly speaking, active inference accounts of depressed mood and negatively valenced affective states centre upon inferences about the uncertainty or volatility of the world and how the body is managed in the face of such uncertainty. Technically, such inferences are conceptualised in terms of the reliability or confidence (i.e., the *precision*) attributed to top-down prior beliefs and sources of incoming sensory evidence [180,181,182]. Physiologically, such inferences are influenced by neuromodulation: neurotransmitters such as dopamine and serotonin modulate the gain of neuronal message-passing to amplify or attenuate information [176,183]. This will occur according to the degree of confidence in one’s generative models, and corresponds psychologically to the selective attention or sensory attenuation of evidence for one’s Bayesian beliefs. According to this framework, negatively valenced states are thought to be associated with increases in uncertainty [184,185,186], prompting an organism to update its prior beliefs by overweighing interoceptive inputs over prior experiences, which has the effect of heightening sensitivity to environmental change [100]. 

So, how does this very general claim relate to depression? A range of proposals have emerged from the literature, which will only be afforded brief attention here. The first distinction to point out is a temporal one—clearly, depression is not just a negatively valenced emotional reaction: it is a pervasive mood state that persists over longer timescales and shapes our affective responses to real-time contexts. Picking up on this, Clark, Watson and Friston have argued that mood states reflect a *hyperprior*, which encodes the expected long-term average of short-term (emotional) fluctuations in precision by determining the set-point of neuromodulatory mechanisms (e.g., dopaminergic and serotenergic systems) that govern the sensitivity of our responses to prediction errors [187]. The central idea here is that neural systems not only maintain estimates of the confidence or precision of their prior beliefs about outcomes, but also estimates of confidence in their beliefs about that precision (i.e., the extent to which the unknowns are known) [119]. In other words, moods determine the precision afforded to the confidence we have in our emotional states. Under this scheme, depression is thought to be associated with a stable prior belief that uncertain or unpredictable outcomes are likely (i.e., low precision beliefs), an expectation that in itself is afforded a high degree of confidence or precision (i.e., there is high confidence in high uncertainty) [119]. This can progress to a chronic, self-perpetuating negative affective state that resists change and can develop into depressive disorder.

Following this logic, depressive disorders have been argued to arise from aberrant interoceptive predictions that originate from the agranular visceromotor cortex [175,188,189,190]. These abnormalities can arise from exposure to sustained distress and generate false predictions about the body’s upcoming autonomic, metabolic and immunological needs that chronically activate physiological stress responses. This, in turn, can result in sickness behaviours such as pervasive negative affect and fatigue, which reduces energy expenditure and can lead to the amotivational and neurovegetative symptoms that characterise depressive disorders [188]. 

Another perspective suggests that depression reflects a self-fulfilling prophecy arising from alterations in top-down expectations that negatively bias predictions and down-regulate reward processing (e.g., [100,113,191]). According to this view, depression is associated with a loss of confidence in descending predictions [192], the dysregulation of precision weighting [193], and overly precise prior expectations for depressive schemas, including strong expectations of self-worthlessness and that the world is uncontrollable [119]. These proposals resemble computational models of reinforcement learning in psychiatry and biology [194,195], suggesting that depression emerges from successive discrepancies between actual and expected reward outcomes (i.e., prediction errors), thereby entrenching (empirical) prior beliefs that rewards are unlikely. This goes on to inhibit reward-approach behaviours. 

Although we agree with these insights, a prevailing issue with these accounts is that they are highly neurocentric: they do not situate the phenotype within the organism’s broader socioenvironmental milieu, which arguably drives such deleterious neurophysiological changes in the first place. Here, we take a leaf from the book of the enactivists by explaining free-energy-minimising processes in terms of the dynamic interplay of body–mind–environment relations [196,197,198]. Previously, we have lent this dynamical perspective a distinctly Darwinian lens, which directs attention to how these depressogenic neurocognitive mechanisms and behaviours have been designed by selection to allow us to adaptively minimise free energy by reducing the volatility or unpredictability of our local social ecology [122]. Why is it that we all have the capacity to become depressed? Surely it must serve some adaptive function? Proponents of active inference are all clearly coming to grips with the various neurobiological mechanisms that underpin depression, but how do these neurocognitive processes result in adaptive changes in our behaviour, which lead to more propitious socioenvironmental changes? Although these models tell us *what* happens in depressed states, they say little about their *adaptive function* or the environmental conditions that produce them. Curiously, though, when compared to other promising models of brain structure and function (e.g., hierarchical predictive processing [83,84]), the *behavioural* component of active inference is particularly unique, not to mention of great interest and utility to psychological scientists. It is surprising, then, that most of the theories on depression have largely neglected this component—beyond emphasising, of course, the downregulation of reward-approach behaviour and other vegetative features of depression, such as sickness behaviours [189]. 

To address this oversight, we have previously leveraged insights gleaned from evolutionary psychology, along with a wealth of supportive findings spanning psychology, psychiatry, and neuroscience, to highlight the key role of social contexts in depression (for other reviews, see [199,200,201]). According to this evolutionary systems perspective, the mild-to-moderate levels of depressed mood that we all experience from time to time reflect an adaptive, socially risk-averse strategy that reduces socioenvironmental volatility when sensory cues suggest an increased likelihood of non-preferred (surprising) interpersonal outcomes, such as rejection, defeat, or interpersonal loss [122]. To achieve this function, the depressive response engenders adaptive changes in perception: it increases the precision afforded to incoming social stimuli, which facilitates perceptual inference and learning about the social world; while also reducing confidence in top-down social predictions, which has the effect of suppressing confident, reward-approach behaviours that run the risk of further exposure to deleterious outcomes (e.g., anhedonia and social withdrawal). Importantly, a unique prediction of this model is that depressed states also generate adaptive signalling behaviours that return the individual to social homeostasis, either by eliciting interpersonal support (e.g., reassurance-seeking) or by defusing conflict with others (e.g., submissive behaviours). We have already provided a proof of concept for this prediction by leveraging active inference to demonstrate these adaptive interpersonal dynamics *in silico* (see [124]), while the capacity of depressive behaviours to elicit interpersonal support from friends and family has long been observed empirically, at least for those exhibiting mild to moderate depressed states [199,202,203]. From this perspective, more severe depression can be seen as a failure to attract interpersonal support or enact other compensatory mechanisms, with excessive reassurance seeking leading to a mutually reinforcing exacerbation of symptoms and risk of rejection [204,205].

Another key distinction between our model and other theories of depression in the active inference literature is that depressed states will not, in the most part, instantiate false inference. Rather, normative depressed states can be thought to reflect adaptive active inference, which cause changes in the self and others that minimise socioenvironmental volatility. It is only when these normative depressogenic mechanisms go astray—typically on account of prolonged socio-environmental stress—that these adaptive patterns collapse into self-perpetuating cycles of false inference and dysfunctional behaviour. 

To elaborate, the model described here furnishes a plausible explanation for how normative depressed states can lead to the sorts of severe, maladaptive depressive responses that are the foci of others in the active inference literature. Namely, when the adaptive depressive response fails, ongoing discrepancies between actual and preferred social outcomes over time (i.e., chronic prediction errors) can entrench aberrant prior beliefs that social rewards are unlikely (e.g., shame, low self-worth, pessimism), which can, in turn, perpetuate risk-averse depressive behaviours (e.g., social withdrawal) and result in a self-fulfilling cycle of dysfunction (e.g., learned helplessness; see [191,206]). From a developmental perspective, this model also accommodates established findings that vulnerability to depressive illness later in life often stems from early exposure to social stress (e.g., abuse or neglect [207,208,209]), on the grounds that early developmental insults are likely to promote prior beliefs that social outcomes are uncontrollable, which heightens the sensitivity of stress response systems to interpersonal stress (e.g., inflammatory immune responses) [122,210]. 

Although this perspective does not sit at odds with other models of depression in the active inference literature, what makes it distinctive is that its focus on the social environment affords unique insights into the free energy minimising properties of depressed states. Importantly, such insights can be translated into empirically testable predictions. For example, one elegant way to test our hypothesis would be to leverage electrophysiological methods that capture prediction error minimisation (e.g., trial-by-trial fluctuations in P300 amplitudes) [211,212] to examine the responses of depressed versus nondepressed individuals who are exposed to unpredictable social (versus asocial) stimuli [122]. The provision of evidence from such designs that depressed individuals respond differently to unexpected social stimuli than non-depressed participants would go some way toward evincing the validity of our hypothesis over its contemporaries. Incidentally, indirect support for this prediction has already been gleaned from similar designs comparing the electrophysiological responses of depressed versus non-depressed participants to unexpected social stimuli (e.g., [213,214]). 

Of course, this model of depression also begs broader research questions, which require sophisticated empirical designs that present an outstanding challenge for active inference theorists. Presuming, for the sake of our argument, that the hypothesis presented here is correct, what are the hierarchical neurocognitive mechanisms responsible for depressive responses to unexpected social stimuli? This question, in itself, can be decomposed into further research avenues. What are the functional roles of the brain regions associated with depression and the patterns of effective connectivity between these regions that serve to minimise social prediction errors? How is the precision of social prediction errors differentially modulated by neurotransmitters like serotonin and dopamine? It is also known that sex differences in susceptibility to depression are strongly associated with hormonal changes and differential brain development in adolescence [215,216]. To what extent are these changes associated with heightened sensitivity to socioenvironmental volatility? Similarly, how do (epi)genetic and developmental influences shape individual differences in the precision weighting of social prediction errors? More broadly still, there have been no explorations in the active inference literature to date on the deleterious effects of neuroticism—a biologically driven, species-typical endophenotype that predisposes people to all sorts of psychopathological outcomes, not just depression [217,218,219]. 

To tackle such issues, greater integration between modelling approaches in active inference and observational and longitudinal methodologies in developmental psychopathology is required to explore the manifold ways in which developmental (and particularly social) contexts influence brain development and bias perceptual inference. To our knowledge, however, there are no existing modelling attempts that directly apply active inference to multiscale, developmental data already collected from real-world samples—such as the Dunedin Multidisciplinary Health and Development study [220] and the Australian Temperament Project [221,222]. In our opinion, methods gleaned from computational psychiatry are likely to hold the most promise when attempting to map such sophisticated patterns across various data sets (also see [123,124]), in order to improve our understanding of the various neurocognitive and behavioural mechanisms that underlie depression—including both its normative, functional states, and the development of its maladaptive manifestations, when such mechanisms go awry. 

Although we are guilty of the same neglect, our analysis here suggests a clear need to test predictions derived from extant models of depression in the active inference literature with available empirical methodologies. As discussed, however, direct empirical assessments of active inference based on real-world data remain surprisingly few and far between [118], no doubt due in part to the barriers that arise when translating abstract informational–theoretic principles into realistic experimental designs based on biophysical, cognitive and/or behavioural observations [166]. Despite its unique explanatory promise, without further empirical progress in this area, the extent to which active inference adds meaningfully to what we already know about depression remains to be seen. 

By now, the discerning reader will have observed that we have yet to discuss whether advances in active inference have resulted in meaningful progress in our attempts to actually help people. Naturally, by increasing our understanding of psychopathology, we stand to develop more targeted and effective methods for prevention, assessment, diagnosis and treatment. But has any of this been translated into clinical practice? Returning to the example of our theory of depression, it is readily apparent that it has clinical implications that have not yet been realised. First, it calls for the need to develop diagnostic and assessment tools that can reliably differentiate socially mediated depressive responses from other depressive outcomes, thereby informing targeted and more effective interventions [122]. Again, we believe that methods born from computational psychiatry are likely to prove key here, but so far, these have yet to eventuate in novel technologies and tools that can be readily implemented in clinical practice. 

Importantly, our theory also has practical implications for the prevention and successful management of depression, which do not stem naturally from other active inference models. By emphasising the importance of the social environment in explaining the aetiology and phenomenology of depression, the model suggests that such efforts should strive to minimise socioenvironmental volatility—by correcting dysfunctional, overly pessimistic top-down interpersonal beliefs (e.g., via CBT [223,224]); by changing behaviours that expose the individual to sources of social uncertainty (e.g., through use of interpersonal psychotherapy to promote adaptive, reparatory responses to social stress; [122]); and/or by altering the individual’s environment to reduce exposure to interpersonal volatility and encouraging engagement with prosocial environments (e.g., through prevention and early intervention efforts that facilitate improvements in social relationships; e.g., [225]). At the same time, this does not preclude other recommendations in the active inference literature that psychopharmacological interventions should be used to adjust erroneous predictions and behaviours stemming from maladaptive, self-perpetuating hyperpriors (e.g., depressed mood), by modulating, for example, the precision afforded to social data [124]. Most antidepressant medications modify the effects of monoamine neurotransmitters such as serotonin and noradrenaline, thereby influencing their modulatory effects on precision weighting and altering the balance between the influence of predictive models versus prediction errors. More recent agents, such as ketamine, also show promise as a means of rapidly affecting brain models directly by altering the excitatory–inhibitory balance enacted by glutamatergic and GABAergic neurons [226]. 

These advances, however, have not sprung from active inference *per se*, but its flexibility and explanatory power has still allowed researchers to map this framework onto such theories and findings, occasionally supporting these models with proof-of-principle simulation studies (which may or may not have real-world validity). So far, then, models applying active inference to psychopathology and its treatment have largely been speculative, with researchers tending to superimpose this theory onto pre-existing models of depression and its treatment. With this in mind, what does active inference bring to the table that makes a unique difference, besides simply explaining why extant theories of mental disorder in psychology and psychiatry, and their best-practice interventions, work so well? 

As discussed, there are also significant translational obstacles facing active inference that have little to do with the veracity of the theory and more to do with its grounding in machine learning and statistical thermodynamics, which are unfamiliar and inaccessible for the majority of psychologists and psychiatrists [166]. Despite the remarkable and rapid advances in computational psychiatry in recent years, paradigms like active inference and reinforcement learning have yet to be translated into readily accessible assessment, diagnostic and treatment tools that clinicians can reliably use in their daily practice. For all their predictive and computational power, one must also wonder whether attempts in computational psychiatry will ever compare to the expert judgement, clinical skills and predictive accuracy of any highly trained and experienced clinician. 

This is a moot point, of course, since approaches in computational psychiatry are geared towards augmenting clinicians’ practice, instead of replacing their expertise. In this vein, we are particularly encouraged by the development of diagnostic technologies that leverage active inference to inform clinical decisions, such as the use of OpenAI Gym to design computer games with diagnostic utility [227]. Otherwise, there is much to be gained from collaborations between active inference experts in computational psychiatry, not to mention those in fields like machine learning and artificial intelligence (e.g., [228,229,230]), and experts in clinical psychology and psychiatry who have already capitalised on technologically advanced clinical tools that assist with assessment, diagnosis and symptom management. A fitting example is the work of Nicholas Allen and his team on a mental health smartphone app designed for young people that tracks their biometric, personal communication, social media and experiential sampling data to map their mental health status in real time, provide support through responsive online therapeutic interventions, and report back to their treating clinicians if these data indicate need or risk [231,232]. Improving these computational techniques with active inference models of psychopathology promises to make a real difference to assessment, diagnosis and treatment on the ground.

Meanwhile, the conceptual reducibility of active inference to the need to minimise uncertainty or stress readily lends itself to clinical applications [185]. Under this transdiagnostic framework, the success of all evidenced-based psychotherapies, and their corresponding cognitive and behavioural techniques, arguably rests on their ability to correct cognitive patterns of false inference and corresponding behaviours that exacerbate socioenvironmental volatility. We have already hinted at existing attempts in the active inference literature to explain the mechanisms of widely endorsed psychotherapies, including the use of CBT (e.g., [224,233]), mindfulness-based cognitive therapy (e.g., [234]); interpersonal psychotherapy (e.g., [122]), and psychodynamic approaches [235,236,237] to correct faulty patterns of perceptual inference, from the top down. Conversely, behavioural approaches offer a viable means to break such patterns by adjusting the feedback from the sensorium and social world to successively reinforce more adaptive expectations about the stability of the individual’s eco-niche, from the bottom up [224]. Notably, the theory has also been applied to the therapeutic alliance itself [238,239,240], suggesting its utility as an explanatory scheme to those who work in clinical practice. Despite such progress, we find it surprising that the traditions in psychology that seem to have the most in common with active inference, particularly control theory and attachment theory, have largely been neglected by the active inference community, despite coming with their own tried and tested psychotherapeutic approaches (e.g., [130,241,242,243,244]). Again, this speaks to a poverty of communication between members of this community and proponents of highly consonant schools in psychology, obstructing the erudition of both. 

That being said, we believe there is already good reason for scientific practitioners to adopt active inference in their clinical practice. First, it promises to inform a therapist’s understanding of their client’s symptoms and the dysfunctional biobehavioural and social patterns they exhibit, along with their choice of therapeutic strategies, to facilitate successful treatment [245]. In this vein, active inference provides a convincing account of the multiple levels through which different treatments operate, which can work directly at the synapse and also by attending to psychosocial factors. Combining treatments that operate at different levels might be the best approach in many cases, with their interactions creating synergies that drive better treatment outcomes. A recent development in depression treatment attests to this. Psychedelic medicines such as psilocybin and LSD activate serotonin receptors directly, inducing profound changes to the sensorium by producing a ‘relaxation’ of the precision weighting of prediction errors [246]. The medication is offered with psychotherapy and has antidepressant effects that emerge during the dosing session and last for weeks afterwards [247]. The active inference framework allows us to make sense of this: the therapy provides a safe and containing setting (reducing social uncertainty), while the psilocybin acts to adjust the precision-weighting of the brain’s predictive models. The more plastic brain is then amenable to embedding the reweighted precisions in the ensuing therapy sessions. 

More ambitiously still, the clear association between psychopathology and socioenvironmental stress speaks to a need to prevent and manage mental ill-health in the broader population by working to resolve uncertainty in community contexts. On the one hand, this reinforces the value of universal, selective and early intervention approaches that effectively prevent conditions like depression by facilitating improvements in social environments [225]. On the other hand, it appeals more broadly to health service design and community development efforts that endeavour to provide secure, socially predictable environments that promote resilience in the face of short-term fluctuations in interpersonal volatility. A clear example here is the design of psychiatric inpatient units that minimise uncertainty by promoting exposure to the sorts of environments favoured by adaptive priors geared towards social inclusion and natural environments. These are just ideas at this stage, but we are sure that many would agree that the evidence-informed explanatory apparatus of active inference is capable of accommodating them and creating new avenues for progressing towards them.

Despite such cause for optimism, it must be said that, to date, the impressive gains active inference has made in our attempts to understanding people have done little to inform our attempts to help them. Beyond our own work with colleagues, we also are unaware of any other contributions in the active inference literature that explicitly adopt an evolutionary systems approach in psychology to inform models of psychopathology and its treatment. As we have attempted to demonstrate in our discussion of depression, this synthetic, multidisciplinary approach offers important insights into our understanding, prevention and treatment of depression that are not afforded by other models in the active inference literature, highlighting the need for greater integration between evolutionary psychology and active inference models in psychiatry. That being said, intradisciplinary fragmentation is endemic to the human sciences [248], a problem that has long been recognised in psychology [151,249]. Compounding matters, the majority of therapeutic approaches in clinical psychology and psychiatry attempt to address proximal causes—namely, dysfunctional neurobiological, cognitive and/or behavioural patterns—while the diagnostic categories upon which such approaches are based are themselves atheoretical [250,251]. The need to address these shortfalls has inspired the growth of complementary fields, such as developmental psychopathology and evolutionary psychiatry [252,253,254,255], although the extent to which they have been taken up by those providing mental health care, let alone proponents of active inference, is very much open to question. Finally, the lack of progress in this area is further complicated by the need for greater theoretical integration between active inference and psychology more generally, as we have already discussed at some length. It is no wonder, then, that applications of active inference to clinical psychology and computational psychiatry also show poor integration with the broader explanatory frameworks proffered by psychology. Indeed, a consistent theme running throughout our discussion is the clear need for greater communication between those who belong to the active inference community and both psychologists and psychiatrists at large. Ultimately, our aim here is to inspire more of these conversations, in the hope of seeing innovative, elucidating and gainful outcomes that ultimately benefit those in need of help.

## 6. Conclusions

When attempting to canvas the influence of a field as burgeoning as active inference in the much broader disciplines of psychology and psychiatry, there are bound to be oversights, and we hope that the field is progressing at a faster pace than we are aware. For our part, we will return to where we began with the two main aims of psychology, and the extent to which active inference has progressed them. There is no question that this theory furnishes unique and powerful conceptual tools that are capable of explaining all patterns of human biohehaviour—tools, no less, that are both neurobiologically plausible and empirically supported models based on mathematical principles [18,58]. In its ability to help us understand people, we contend that active inference takes us one step further than evolutionary psychology, providing a new paradigmatic candidate that is capable of incorporating its insights, while also integrating developmental and more proximate, biopsychosocial explanations for cognition and behaviour. By the same token, active inference is a non-substantive, formal theory of phenotypic mechanics, which demands recourse to substantive research across psychology to inform these content-free models with evidence-based theories that describe how particular patterns of surprise-reducing policies manifest in humans. Here, evidence-informed evolutionary hypotheses are likely to prove key to understanding broadly generalisable, species-typical human traits, while analysing the complex ways in which active inference manifests over the course of ontogeny requires insights gleaned from developmental psychology and psychopathology. In our view, the challenge here is to transcend translational obstacles and disciplinary divisions by calling for greater collaboration between proponents of active inference and psychological scientists to furnish the former with process theories of cognition and behaviour grounded in real-world empirical evidence, and the latter with a biologically plausible, formal theory of all biobehavioural patterns—psychopathological or otherwise. Arguably, the need for greater integration between active inference and research in psychology remains as alive today as it was over a decade ago. Still, progress in the field is swift and quickening, and we hope that it is only a matter of time before we see a genuine change in the metatheoretical landscape of psychology that synthesises the two.

Having said that, we will close by returning to the second main aim of psychology—has the theory of active inference actually helped people? To date, it is fair to say that it has achieved very little—owing in large part to its technical inaccessibility but also to the slow and steady pace of research in clinical psychology and psychiatry. It took many years for Beck and colleagues to accrue the necessary clinical evidence behind CBT before its ubiquitous uptake in mental health care, the development of tolerable and effective psychotropics for severe mental disorders is still in progress, and any new form of psychotherapy has to be manualised and supported by sufficient clinical trials to be broadly taken up by practitioners. As such, progress in clinical applications of active inference is likely to take some time. In our opinion, the most immediate and promising opportunity centres on combining active inference approaches in computational psychiatry [163,182] with extant digital technologies for mental health care, such as the effortless assessment of risk states tool [231]. More ambitiously still, there is genuine potential for active inference to inform models of mental health service design, community development and large-scale social programs, by emphasising the need to sculpt our institutions around the human need for social homeostasis and environmental certainty. Despite their ambitiousness, we believe that active inference holds enough promise to see such developments eventually come to light.

## Data Availability

No new data were created or analyzed in this study. Data sharing is not applicable to this article.
